# Global point-of-care ultrasound education and training in the age of COVID-19

**DOI:** 10.1186/s12245-021-00338-9

**Published:** 2021-02-18

**Authors:** Onyinyechi F. Eke, Patricia C. Henwood, Grace W. Wanjiku, Abiola Fasina, Sigmund J. Kharasch, Hamid Shokoohi

**Affiliations:** 1 Department of Emergency Medicine, Massachusetts General Hospital, Boston, MA USA; 2 Department of Emergency Medicine, Sidney Kimmel Medical College - Thomas Jefferson University, Philadelphia, PA USA; 3 Department of Emergency Medicine, Brown University, Providence, RI USA; 4 Emergency Healthcare Consultants, Lagos, Nigeria

**Keywords:** Global point-of-care ultrasound, COVID-19, Education, Training

## Abstract

The COVID-19 pandemic has disrupted traditional global point-of-care ultrasound (POCUS) education and training, as a result of travel restrictions. It has also provided an opportunity for innovation using a virtual platform. Tele-ultrasound and video-conferencing are alternative and supportive tools to augment global POCUS education and training. There is a need to support learners and experts to ensure that maximum benefit is gained from the use of these innovative modalities.

## Introduction

Point-of-care ultrasound (POCUS) offers a readily available and relatively low-cost tool to diagnose and manage medical conditions in resource-limited settings where imaging modalities are scarce and cost-prohibitive [1]. POCUS education is also one of the competency milestones for emergency medicine training increasingly globally, with skill requirements in place in the USA, Haiti, Rwanda, Ethiopia, and Ghana [2–6].

Innovation in POCUS training and education has surged as a prime focus for many global emergency care improvement efforts. Global POCUS education and training typically involves the creation of programs in low- and middle-income countries (LMIC) with the goal of building local capacity [1, 7]. Most training programs involve planned didactic sessions with learners at different levels of training: physicians, residents, nurses, medical officers, or other advanced practice providers. Didactic learning is then accompanied by hands-on scanning sessions. However, in LMIC, there is often insufficient training, inadequate access to ultrasound machines, lack of guidance when scanning, and lack of trainee feedback [8].

The COVID-19 pandemic has disrupted every sector of global health training, including on-site education and faculty exchange. In LMIC, the impact of the pandemic is even more deleterious as many of these economies were already on the brink of economic recession prior to the pandemic. Therefore, individuals and institutions commencing or continuing international educational and training programs will have to adapt and innovate in this period of heightened global challenge. In this brief report, we explore and highlight alternatives, including tele-ultrasound and video-conferencing, as readily and widely available resources.

## Tele-ultrasound

Traditionally, ultrasound education for physicians and other emergency care providers includes a combination of didactic lectures and case/image reviews, combined with hands-on image acquisition and interpretation by trainees. This is ideally followed by a period of quality assurance in regard to image review and interpretation. Prior studies have shown the feasibility of  implementing POCUS training and the improvement of skills with repeated training [9].

Tele-ultrasound allows learners performing bedside ultrasound to receive instruction and feedback from instructors at different geographical sites. The tele-ultrasound guidance can be given asynchronously and synchronously. There has already been successful implementation of tele-ultrasound and tele-mentoring to guide POCUS education, training, and patient management [10, 11]. In a study by Choo et al., tele-mentoring was used to guide the acquisition of psychomotor skills in ICU nurses without prior POCUS experience [12]. In another study by Smith et al., it showed that cell phones were a viable tele-ultrasound option to educate novice learners [13]. Tele-ultrasound platforms include cell phones using Facetime (Apple Inc., Cupertino, CA, USA) or WhatsApp ( Facebook Inc., Menlo Park, CA, USA), IIT’s Reacts platform (Innovative Imaging Technologies) through Philips Lumify and Butterfly TeleGuidance through Butterfly IQ (Butterfly Network Inc., Guilford, CT). The presence of artificial intelligence (AI) and augmented reality (AR) yield opportunities to optimize image quality and provide real-time guidance on probe placement; these are newer features on specific platforms in addition to image interpretation support that increase trainee learning while reducing the spread of infection [14].

To augment tele-ultrasound in LMIC, there has to be facilitation of internet connectivity and/or cell phone usage. There is ubiquitous use of cell phones in LMIC particularly, in African countries where most people use cell phones to access the internet. Therefore increasing cell phone usage in training centers would likely lead to increased potential interaction with tele-ultrasound platforms.

The challenge remains that internet access through cell phones, while often more stable than computer-based internet, is expensive and cost-prohibitive. The other tele-ultrasound platforms and software require a stable and strong internet connection to function effectively. This cost can be subsidized by trainee and expert institutions as well as manufacturers of the tele-ultrasound platforms. An additional challenge is trainees in LMIC countries often have heavy clinical loads and may not have adequate time to engage in tele-mentoring sessions. There needs to be protected educational time for trainees and incentives to bolster trainee engagement.

Lastly, the success of these programs may be improved by remote POCUS experts and mentors available for “on call” tele-ultrasound consults. This availability often hinges on support for this non-clinical responsibility.

## Video-conferencing

With hands-on training that can be supervised remotely by tele-ultrasound, there is also a need for didactic education for learners. With current COVID-19 travel restrictions, it is reasonable to ask how teachers and trainees separated by significant distance initiate or continue POCUS didactic education. We offer an example of how video-conferencing can be implemented in a format that engages learners and experts.

In May 2020, the emergency ultrasound division at Massachusetts General Hospital created a virtual global ultrasound conference. Using ZOOM (Zoom Video Communications, Inc., San Jose, CA), a video-based conferencing platform, a 1-month program with 3-h sessions every Wednesday was organized. Each week, invited experts from different parts of the world spoke about the state of emergency medicine, COVID-19, and POCUS in different settings. Many of the speakers are pioneers in their field working in Uganda, Kenya, Nigeria, Thailand, Bolivia, Israel, and the USA. These experts were involved in active COVID-19 efforts on an institutional and national level, device manufacturing, health policy advocacy, and emergency medicine education and training. There was also a journal club with authors of selected articles invited to discuss their papers. The journal club topics included tele-ultrasound in resource-limited settings, POCUS implementation, barriers in emergency training programs globally, and the effect of remote quality assurance feedback in LMICs. Learners were also a diverse group from several continents, all active in contributing to discussion.

The advantage of video conferencing was the opportunity to discuss how different institutions and communities were faring at the peak of the current crisis, the formation of new partnerships, communal learning, and the cost-savings as learners and experts did not need to travel to have this educational experience.

After the global ultrasound conference, video conferencing is still being used to teach scanning techniques, review ultrasound images, and discuss specific POCUS topics. The ability to record these instructional videos and archive for future use by learners is also one of the advantages of video conferencing. Learners from LMIC can access these videos asynchronously to augment POCUS education.

The biggest challenge using the ZOOM platform for didactic education with a global audience was internet instability, as a result of limited bandwidth particularly for international experts and learners. Sometimes, this problem was mitigated by having the live-video feature turned off. Other challenges included scheduling speakers in different time zones; this was resolved by scheduling speakers well in advance and asking for their preferred dates and times with the audience being flexible with timing.

Programs interested in expanding global POCUS education or exposure may consider this framework to build an educational experience that connects educators and trainees around the world (Table 1).

**Table 1:** Global POCUS educational innovations in COVID-19

Modality	Advantages	Challenges	Recommended solutions
Tele-ultrasound	● Synchronous and asynchronous learning and feedback for trainees ● Multiple platforms for usage including but not limited to cell phones, Philips Lumify IIT’s Reacts platform, Butterfly TeleGuidance ● Use of artificial intelligence (AI) and augmented reality (AR) for real-time guidance on probe placement and optimizing image quality	● Cost of internet and software for tele-ultrasound ● Baseline heavy workload on trainees ● Lack of time support for remote faculty	● Subsidize stable and reliable internet service to trainees including cell phone service and subscription to tele-ultrasound platforms ● Consider low broad-band alternatives for tele-ultrasound ● Provide protected educational time for trainees and incentivize learning ● Provide faculty support, e.g., protected time to engage in tele-consults
Video-conferencing	● Cost saving for experts and learners including no need to travel ● Recorded instructional and didactic videos ● Communal bidirectional learning and formation of new partnerships	● Unstable internet ● Different time zones between experts and learners	● Subsidize stable and reliable internet ● Consider low-cost web-based conferencing ● Turn off live-video feature during live sessions for low broad-band connections ● Advanced scheduling of speakers asking for date and time preferences

## Further considerations

The impact of COVID-19 will be ongoing as the efforts to develop and distribute effective vaccines are in still progress, even when travel restrictions are lifted, and countries open their borders [15]. The pandemic has already changed how we perform POCUS locally in the USA. Consideration has to be given to the usage of scarcely available personal protective equipment, lack of adequate testing for patients, and machine cleaning protocols considering SARS-CoV-2 fomite data [16]; the use of POCUS is not without risk.

With the global financial crisis, the reality looms that there might be even less funding sources available for global health projects including POCUS training and education. This includes the cost of travel, lodging, machines, training staff, educational materials, internet, and cloud-based sharing services among other expenses. Thought must be given to other sources of funding including private organizations, government, and local authorities at international sites. Apart from the financial and economic impact of COVID-19 on many LMIC, the effect on medical education on already strained health care systems may have significant consequences that outlive this pandemic.

## Conclusion

The COVID-19 pandemic has disrupted global POCUS education and training; however, it has created an opportunity for innovation and collaboration using a virtual platform. More research will be needed to follow-up on the effectiveness and sustainability of a virtual platform for partnerships in global health and POCUS education. Considerations for patient and clinician safety, as well as ongoing funding needs, will still require attention as travel comes back into play. However, we believe adopting these new paradigms as methods of providing global health education may help overcome many obstacles in the long run. Therefore, many of these new methods can and should be included in traditional global POCUS educational methods moving forward.

## Data Availability

Not applicable

## Abbreviations

COVID-19: Coronavirus disease 2019
POCUS: Point-of-care ultrasound
LMIC: Low- and middle-income countries
ICU: Intensive care unit
SARS-CoV-2: Severe acute respiratory syndrome coronavirus 2
